# The Role of the Endothelium during Antibody-Mediated Rejection: From Victim to Accomplice

**DOI:** 10.3389/fimmu.2018.00106

**Published:** 2018-01-29

**Authors:** Amy Rachael Cross, Denis Glotz, Nuala Mooney

**Affiliations:** ^1^INSERM U1160, Paris, France; ^2^University Paris Diderot, Sorbonne Paris Cité, Paris, France; ^3^AP-HP, Hôpital Saint Louis, Département de Néphrologie, Paris, France; ^4^LabEx Transplantex, Université de Strasbourg, Strasbourg, France

**Keywords:** endothelial cells, antibody-mediated rejection, donor-specific antibodies, transplantation immunology, inflammation

## Abstract

Antibody-mediated rejection (AMR) of solid organ transplants is characterized by the activation and injury of the allograft endothelium. Histological and transcriptomic studies have associated microvascular inflammation and endothelial lesions with the severity of rejection and poor graft outcomes. The allograft endothelium forms the physical barrier between the donor organ and the recipient; this position directly exposes the endothelium to alloimmune responses. However, endothelial cells are not just victims and can actively participate in the pathogenesis of rejection. In healthy tissues, the endothelium plays a major role in vascular and immune homeostasis. Organ transplantation, however, subjects the endothelium to an environment of inflammation, alloreactive lymphocytes, donor-specific antibodies, and potentially complement activation. As a result, endothelial cells become activated and have modified interactions with the cellular effectors of allograft damage: lymphocytes, natural killer, and myeloid cells. Activated endothelial cells participate in leukocyte adhesion and recruitment, lymphocyte activation and differentiation, as well as the secretion of cytokines and chemokines. Ultimately, highly activated endothelial cells promote pro-inflammatory alloresponses and become accomplices to AMR.

## Antibody-Mediated Rejection (AMR)

With the decline in the incidence of acute rejection, chronic rejection has become the major cause of allograft loss in solid organ transplantation (1). Moreover, AMR represents the predominant mechanism of chronic rejection (2, 3). In the case of renal transplantation, AMR is identified by the presence of circulating donor-specific antibodies (DSAs), morphologic evidence for tissue injury such as transplant glomerulopathy (characterized by reduplication of glomerular basement membranes), and evidence for antibody interaction with the vascular endothelium such as C4d deposition and/or microvascular inflammation (4, 5). Similar criteria for AMR diagnosis are used in cardiac (6), pulmonary (7), and pancreatic (8) transplantation and establish the involvement of the endothelium in the processes of AMR.

Both DSA existing prior to transplantation and DSA produced *de novo* post transplantation are harmful, although a recent study reported that AMR patients with preexisting DSA had superior graft survival to patients with *de novo* DSA (9). In the subset of patients with *de novo* DSA, the detection of endothelial lesions was associated with the poorest survival rates. While the correlation between DSA and microvascular endothelial lesions is well recognized, recent data have revealed that DSAs also accelerate arteriosclerosis (10) and that they have a key role in premature and accelerated fibrosis of the allograft (11).

In AMR, the vast majority of known DSA are directed against the highly polymorphic HLA antigens. DSAs directed against either class of HLA antigen are deleterious; however, DSAs directed against HLA class II antigens have been more strongly associated with late-onset AMR, *de novo* antibody production, and reduced graft survival (12). Patients who develop HLA class II DSA have a higher risk of developing transplant glomerulopathy and microvascular endothelial damage associated with reduced graft survival (13). Of note, DSAs do not exclusively target HLA antigens; several non-HLA DSAs have been identified in proteomic and transcriptomic studies following allograft rejection (14). Such non-HLA DSAs often target endothelial-expressed antigens and accelerate vascular injury, such as vimentin, MICA, collagen, laminin-like globular domains of perlecan, and the angiotensin receptor type 1 (15, 16).

Endothelium damage in the presence of DSA was first considered to be a consequence of complement activation on the basis of the detection of a product of the activated complement cascade, C4d, in the microvasculature of AMR graft biopsies. Following studies on animal and human tissues, it became clear that complement activation is not a prerequisite for allograft vasculopathy (17, 18). Nonetheless, complement-binding anti-HLA DSAs have been associated with an increased rate of AMR, worse tissue damage, and more extensive microvascular inflammation than non-complement-binding anti-HLA DSAs (19).

These data demonstrate the clear involvement of the endothelium in AMR. Furthermore, they associate DSA and even complement with increased endothelial cell inflammation and vascular damage.

## Endothelium Activation in AMR

The allograft microvasculature is the initial site of contact between the recipient’s circulating immune system and donor antigens. As such, the endothelium takes on the role of a primary target of alloresponses. However, the endothelium is not an inert structure and actively participates in vascular and immune homeostasis. AMR-associated inflammation, alloantibodies, and activation of the complement cascade have been shown in recent studies to produce distinct endothelial phenotypes and impact their capacity to regulate and activate the alloimmune response (20–22).

Comparison of gene transcripts isolated from DSA-positive and -negative patient biopsies has confirmed endothelium activation as a characteristic of AMR (23). Hidalgo et al. reported a set of 132 DSA-specific transcripts that were functionally associated with HLA, interferon gamma effects, macrophages, natural killer (NK) cells, endothelial cells, inflammation, and immunoglobulins. Furthermore, 23 transcripts were selectively expressed during rejection from DSA-positive patients and they were chiefly expressed by the endothelium (8/23). This evidence supports the notion that DSAs contribute to a distinctive activation of endothelial cells during rejection.

The allograft endothelium is capable of expressing HLA molecules, which in the context of allotransplantation exposes the endothelium to recognition by the recipient’s humoral and cellular immune system. The expression of HLA class I antigens is readily detected in the renal microvascular endothelium, whereas that of HLA class II antigens is modest in the steady state and significantly increased in rejection (24, 25). HLA class II antigen expression is regulated by pro-inflammatory factors and displays a hierarchical expression of its isotypes (DR > DP > DQ) (26) and a non-identical time course of expression with HLA-DR and −DP being more readily induced than HLA-DQ. HLA-DR and HLA-DQ are frequently the target of *de novo* DSA and are significantly upregulated by the microvasculature post transplantation, as well as in AMR (27, 28).

Signaling mediated by ligand binding to HLA molecules in different cell types has been extensively reviewed elsewhere (29–31). In the endothelial cell, the alloantibody binding of endothelial HLA class I and II molecules has been shown to mediate signal transduction, independent of complement activation ((20, 21) and reviewed in ref. (32)). The relevance of these signaling pathways has been confirmed by the detection of phosphorylated-signaling proteins, such as S6RP, in biopsies from patients with cardiac allograft vasculopathy and their quantitative correlation with AMR, but not other forms of rejection (33). Non-HLA DSAs directed against the angiotensin receptor type 1 DSA have been reported to be biologically active in vascular cells through the activation of Erk, AP-1, and NF-kB activation (34).

Complement activation, as a result of DSA binding, can synergize with the direct signal transduction induced by DSA-HLA ligation on the endothelium to further modify endothelial activation. This synergy has been observed to increase exocytosis of pro-thrombotic and pro-inflammatory molecules. While DSA-activated exocytosis was independent of complement activation, it could be increased by the addition of the C5a fragment of complement (35). Also, panel-reactive antibodies and the complement membrane attack complex combine to amplify a set of pro-inflammatory genes and the molecular expression of VCAM-1 (21).

In this review, we will use recent studies to explore endothelial cell participation in the physiopathology of AMR. We will primarily address atypical endothelial activation in the allograft and how these conditions may dictate alternative interactions with the cellular effectors of AMR, such as lymphocytes, NK cells, and myeloid cells.

## Activated Endothelial Cells Modulate Allogeneic Lymphocytes

As antigen-presenting cells, albeit non-professional, endothelial cells are in a unique position to promote the recognition of “non-self” allograft antigens, to recruit leukocytes and to modulate alloimmunity. Leukocyte infiltration is typical during allograft rejection. Interestingly, the composition of the infiltrates has been observed to contain equivalent numbers of intravascular CD3+ lymphocytes and CD68+ myeloid cells in the biopsies from cardiac-transplant patients with AMR (36).

Under inflammatory conditions, endothelial HLA molecules and adhesion molecules are upregulated and are sufficient to activate the alloproliferation of CD4+ T lymphocytes and concomitant increases in IL-2 and IFNγ secretion (37, 38). In an *in vitro* experimental model, allogeneic human ECs expressing HLA-DR were capable of selectively activating CD4+ T cell differentiation toward the pro-inflammatory Th17 and the anti-inflammatory Treg subsets, by mechanisms involving IL-6 secretion and CD54 expression, respectively (38). Allograft infiltration of Th17 cells has been associated with shorter allograft survival (39), while the proportion of intragraft Treg cells has been positively correlated with graft survival (40, 41). The importance of HLA class II was also underlined by a study wherein the blocking of expression of endothelial HLA class II antigens by antibodies or siRNA was able to prevent CD4+ T activation and the IL-2 production required for a CD8+ alloresponse (42). These data led to the proposal that the extent of endothelial expression of HLA class II has functional consequences on lymphocyte-associated alloresponses.

**Figure 1:**
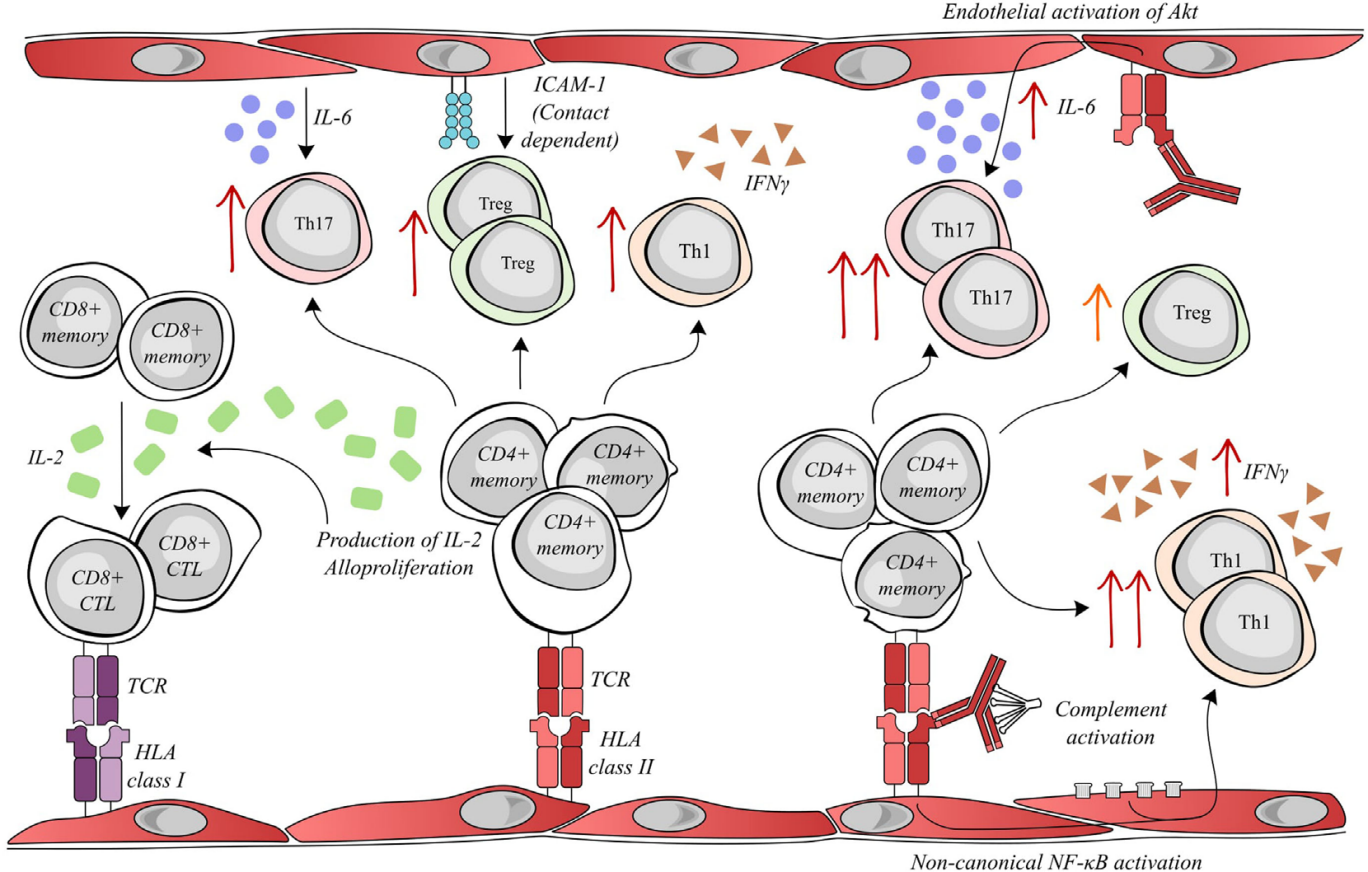
Activated endothelial cells modulate allogenic T lymphocyte activity. Allogenic endothelial cells expressing HLA class II molecules are capable of inducing the secretion of IL-2 and alloproliferation by CD4+ memory lymphocytes. The T helper cell production of IL-2 facilitates the differentiation of memory CD8+ T cells into cytotoxic lymphocytes (CTLs), which can mediate rejection by targeting donor HLA class I molecules. Meanwhile, alloproliferation in CD4+ lymphocytes is associated with the selective expansion of pro-inflammatory Th17 (endothelial IL-6-dependent), anti-inflammatory Treg (endothelial ICAM-1 dependent) and pro-inflammatory Th1 subsets. Interestingly, donor-specific antibodies (DSAs) binding to HLA class II molecules alter endothelial cell immunogenicity. DSA binding can activate the Akt/PI3K-signaling pathway, consequently increasing endothelial IL-6 secretion and increasing the expansion of the pro-inflammatory Th17 subset. In addition, the amplification of the Treg population was decreased after DSA binding. Both the binding of DSA and the sub-lytic activation of the complement cascade can synergize to activate non-canonical NF-κB signaling in endothelial cells, ultimately resulting in an increased expansion of the Th1 subset and a greater secretion of IFNγ.

In our most recent study, HLA class II antibody binding to activated endothelial cells, in an inflammatory context, induced the phosphorylation of Akt, MEK, and ERK. The downstream effect of HLA class II ligation was modified by endothelial immunogenicity. The activation of Akt by anti-HLA-DR antibody was implicated in the increased IL-6 secretion by the endothelial cells in an allogeneic setting and therefore enhanced endothelial cell-mediated differentiation of pro-inflammatory Th17 (20). Jane-Wit et al. showed that panel-reactive antibodies activated an inflammatory gene expression profile in endothelial cells through the activation of the non-canonical pathway of NF-kB. This signal transduction increased endothelial cell immunogenicity and promoted CD4+ -T cell differentiation toward the pro-inflammatory Th1 subset. Antibody-mediated increases in endothelial cell immunogenicity were significantly enhanced by the activation of the classical complement cascade (21) (see Figure 1).

Regarding costimulatory molecules, ICOSL is strongly expressed by human endothelial cells in the presence of TNFα and IL-1β and played a functional role in CD4+ T activation (43). There are discrepancies in the literature regarding PD-L1; vascular endothelial expression was required for the generation of allogeneic Treg in a mouse-transplant model (44), whereas the lack of endothelial PD-L1 expression resulted in a greater generation of effector T cells and cardiac-transplant rejection in a chimeric mouse model (45). PD-L1 did not appear to be implicated in Treg expansion in an experimental model of human microvascular EC (38).

## The NK Cell Contribution to Endothelium Damage

Strong support for the implication of NK cells in AMR came from a mouse model of cardiac transplant in which the adoptive transfer of DSA led to chronic allograft vasculopathy and in which the depletion of NK cells limited cardiac allograft vasculopathy (46). Further support for the implication of NK cells in AMR in patients also came from the initial study by Hidalgo et al., which revealed that 6/23 transcripts selectively associated with the presence of DSA were highly expressed in NK cells (23). The data from transcript studies were supported by the detection of CD56 + cells (alongside CD68 + cells) in peritubular capillaries of biopsies from patients with AMR. Further study indicated that high NK transcript expression was associated with late-stage AMR, with microvascular inflammation, and with DSA (3). The notion of IFNγ release by the NK cell, following triggering by DSA fixation to both the EC and the NK cell, heightening local inflammation, which in turn would increase both HLA class I and II molecular expression by the endothelium and thus enrich the density of targets for DSA binding, was raised. While high NK transcript expression is associated with AMR, they can also be detected in biopsies from patients with T cell-mediated rejection.

**Figure 2:**
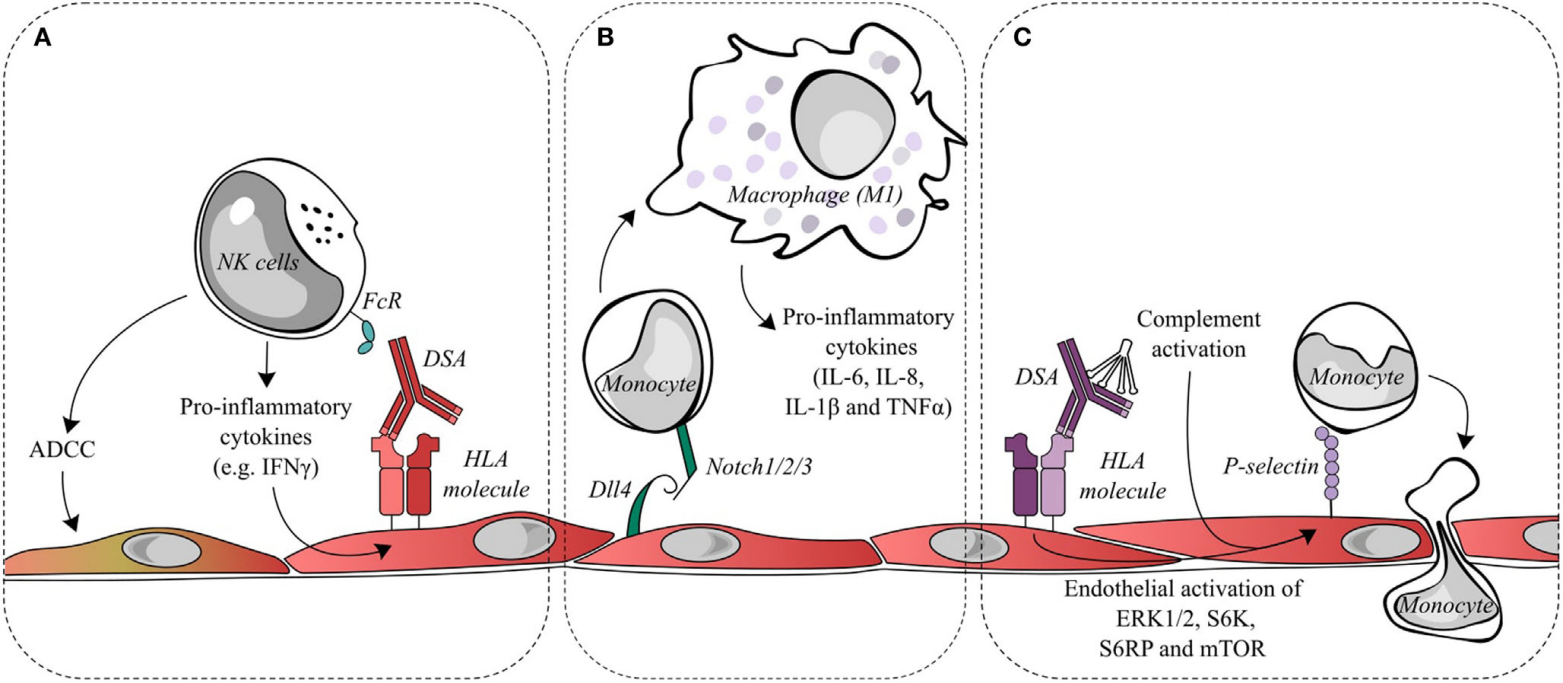
Endothelial cell activation and recruitment of natural killer (NK) cells and monocytes. **(A)** Donor-specific antibodies (DSAs) binding to endothelial cells may lead to the ligation of Fc receptors on NK cells and contribute to antibody-dependent cell-mediated cytotoxicity (ADCC) directed against the allograft or to the release of pro-inflammatory cytokines, such as interferon gamma (IFNγ). IFNγ is a key factor in the quantitative expression of HLA class II molecules. Changes in IFNγ could conceivably increase the targets for DSA and thus increase antibody-mediated damage. **(B)** During antibody-mediated rejection, Dll4 is upregulated on endothelial cells and is capable of modulating the differentiation of monocytes into macrophages displaying a pro-inflammatory M1 phenotype. **(C)** DSA binding to endothelial HLA class I molecules initiates intracellular signaling, such as the activation of ERK1/2, S6 kinase, S6 ribosomal protein, and mTOR. This signal transduction leads to the exocytosis of Weibel–Palade bodies and increases cell surface P-selection, which can augment monocyte recruitment and adhesion to the endothelial layer. Classical complement activation, resulting from DSA binding, is capable of synergizing with DSA signaling to further upregulate endothelial cell surface P-selectin and increase monocyte recruitment.

The role of NK cell interactions with the graft endothelium in AMR has also been postulated as a mechanism of allograft damage. Such interactions were proposed to take place after DSA binding to the endothelium and the simultaneous binding of the constant antibody fragment (Fc) to circulating NK cells. Several outcomes of antibody binding to NK cells through activating receptors are possible, including antibody-dependent cell-mediated cytotoxicity (ADCC) with target cell lysis or the release of pro-inflammatory cytokines. The latter is a more likely consequence in AMR as lytic damage to the endothelium is infrequently observed (see Figure 2A).

A recent study set out to model the DSA interaction with the NK cells *in vitro* by stimulating human NK cells from healthy donors with an immobilized antibody directed against CD16a (47). Interestingly, the detection of both chemokines and inflammatory cytokines supported the idea that NK cytokine production may be more relevant than cell lysis to AMR. Although the six NK-associated transcripts initially described by Hidalgo et al. (23) were expressed, they were not increased by antibody binding to NK cells.

A novel functional assay of NK activity was recently tested using DSA and NK cells from ABMR patients to indirectly determine NK lytic function and the cytotoxic potential of patient alloantibodies, the NK Cellular Humoral Activation Test (NK-CHAT) (48). Results from the study showed that ADCC activity was lower in transplant recipients with preserved graft function and that scoring the outcome of the NK-CHAT test allowed the prediction of ABMR.

## Endothelial Cell Recruitment and Polarization of Monocytes/Macrophages

Immunohistochemical studies of rejecting biopsies have observed significant leukocyte infiltration associated with the severity of allograft damage. A majority of these cells are intravascular CD68+ monocytes/macrophages (49, 50). In biopsies from cardiac-transplant patients with AMR, an equivalent number of intravascular CD3+ lymphocytes and CD68+ myeloid cells were observed (36). Transcriptomic studies of biopsies have identified macrophage transcripts as the selective genes set that allow the discrimination of AMR and non-AMR patients (51). The myeloid gene set correlates with both the severity of AMR and the MFI of anti-HLA DSA. Furthermore, the changes in macrophage-associated genes suggest Fcγ receptor-mediated phagocytosis as an active pathway in AMR (51).

Monocyte and macrophages may be important mediators of rejection through different mechanisms including antigen processing and presentation, co-stimulation, cytokine production, and tissue remodeling (52). Endothelial cells interact with monocytes through cytokine crosstalk as well as contact-dependent mechanisms, and recent research has elucidated specific mechanisms by which AMR-activated endothelial cells may act upon monocyte responses.

Reed et al. found that HLA class I antibodies are capable of activating endothelial cells by an increase in intracellular calcium, leading to the exocytosis of Weibel–Palade bodies and a concomitant increase in cell surface P-selectin (53). This increase in P-selectin is sufficient to modulate and increase monocyte adhesion to the endothelial cells, and a mouse model of anti-HLA class I DSA-induced AMR found that blocking P-selectin significantly reduced the macrophage load in the graft (54). Furthermore, following the increase in surface P-selectin, Fc receptors binding to the HLA class I-bound DSA contributed to the stability of monocyte adhesion (55). Additionally, the activation of the classical complement cascade by the Fc portion of DSA that releases components such as C5a and C3a has been shown to further enhance endothelial surface P-selectin increases mediated by HLA class I DSA and thereby increase monocyte recruitment to the endothelium (56) (see Figure 2C).

Notch ligands and receptors are important regulators of immune cells. Within endomyocardial biopsies, the Notch4 receptor is exclusively expressed on the apical side of endothelial cells and is downregulated during AMR. Conversely, the Notch ligand Dll4 is detected and upregulated on both intravascular macrophages and endothelial cells during AMR. Pabois et al. found that the pro-inflammatory stimulation of endothelial cells *in vitro* could recreate the changes in Notch4 and Dll4 observed in AMR (22). Endothelial Dll4 regulates Notch signaling in monocytes and encourages the differentiation of monocytes into macrophages with a predominantly pro-inflammatory M1 phenotype, which corresponds to the phenotype of intravascular macrophages observed in the endomyocardial biopsies during AMR. The M1 subset is considered to be phagocytic and pro-inflammatory, while the M2 subset is attributed to anti-inflammatory and pro-fibrotic abilities (see Figure 2B).

## From Victim to Accomplice

The endothelium has been long recognized as a victim in AMR. Endothelial lesions, endothelial cell swelling, and reduplication of the capillary basement membranes are strongly associated with the severity of AMR and poor graft outcomes.

However, studies have only recently begun to reveal the active involvement of endothelial cells in the physiopathology of rejection. Allotransplantation and AMR expose the endothelium to inflammation, HLA/non-HLA DSA, and often complement activation. As a consequence of such stimulation, endothelial cells become atypically activated with an altered immunogenicity and an altered capacity to modulate alloimmune responses. DSA-bound endothelial cells act as a scaffold for Fc-dependent NK cell activation. Activated endothelial cells can induce the alloproliferation of CD4+ T lymphocytes, alter T cell polarization, and indirectly aid anti-graft CD8+ T cell responses. Activated endothelial cells possess a greater capacity for monocyte recruitment and are implicated in pro-inflammatory monocyte differentiation.

Is endothelial cell activation a consequence or a cause of AMR? Endothelial cell activation is associated with episodes of AMR, but this does not exclude the possibility that the activation precedes the clinical diagnosis. For example, HLA-DQ expression is upregulated post transplantation in stable as well as in deteriorating allografts, despite the molecule being regulated by inflammation (27, 28). The idea of a subclinical activation of the endothelium could contribute to the chronicity of AMR through long-standing low-level activation of T cells, NK cells, and myeloid cells despite the systemic therapeutic immunosuppression. Few studies have assessed the impact of such therapies on the immunomodulatory properties of the allograft endothelium (57) and fewer still have tried to directly manipulate the endothelium. Yet, with the growing understanding of endothelial involvement in the mechanisms of AMR, this avenue for treatment remains to be explored.

## Author Contributions

AC, DG, and NM contributed to the writing of this review.

## Conflict of Interest Statement

The authors declare that the research was conducted in the absence of any commercial or financial relationships that could be construed as a potential conflict of interest.
